# Highly selective sensing of tetracycline by fluorescent carbon dots derived from spent coffee grounds *via* a green microwave route

**DOI:** 10.1039/d5ra07629c

**Published:** 2026-01-01

**Authors:** Haochen Shen, Ying Chu, Ziyi Liu, Chuhan Zhang, Yang Yu, Shaohui Yang

**Affiliations:** a School of Environmental Science and Engineering, Tianjin University Tianjin 300354 China shaohuiyang77@tju.edu.cn; b Teda Greening Science and Technology Group Co., Ltd Tianjin 300457 China

## Abstract

Tetracycline (TC), a widely used broad-spectrum antibiotic, can accumulate in ecosystems through environmental release, posing risks to ecological security and human health. This study presents a green and efficient microwave-assisted method for synthesizing fluorescent carbon quantum dots (B-CQDs) using spent coffee grounds as a precursor. The as-prepared B-CQDs were employed as a fluorescent probe for the highly selective detection of TC in water. Characterization results revealed that the B-CQDs possess good water dispersibility, high fluorescence stability, and a relatively high quantum yield of 11.2%. Under optimal conditions, B-CQDs exhibited a highly selective fluorescence quenching response towards TC, with a linear detection range of 0–140 µmol L^−1^ and a low detection limit (LOD) of 0.36 µmol L^−1^. Mechanistic investigations indicated that the quenching process is primarily attributed to the synergistic effect of the inner filter effect (IFE) and static quenching. The practicality of the method was demonstrated by analyzing TC in real water samples (tap water, lake water, and swine farm wastewater), achieving satisfactory spike recoveries of 98.8–105.5% with relative standard deviations (RSD) below 3.53%, confirming its good accuracy and practical applicability. Furthermore, the greenness of the proposed method was evaluated using the Greenness Evaluation Metric for Analytical Methods (GEMAM) system, yielding an excellent comprehensive score of 8.536 out of 10, which underscores its superior environmental friendliness. This work not only demonstrates a pathway for the valorization of waste biomass but also provides a green detection methodology and a potential high-performance sensing material foundation for the environmental monitoring of tetracycline antibiotics.

## Abstract 2

Green-synthesized carbon dots (B-CQDs) from coffee grounds detect tetracycline *via* concentration-dependent fluorescence quenching. They offer sensitive, selective, and eco-friendly analysis in complex water matrices.

## Graphical abstract

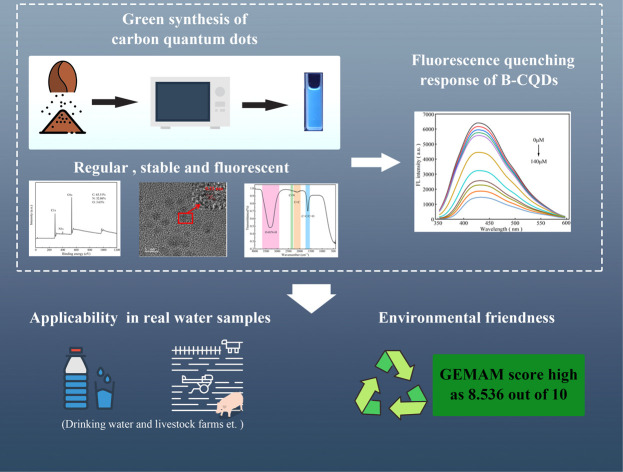

## 1. Introduction

Tetracycline (TC), a broad-spectrum antibiotic, is extensively utilized in human medicine, animal husbandry, and aquaculture owing to its potent antibacterial activity, cost-effectiveness, and favorable absorption characteristics.^1,2^ As the world's largest producer and consumer of antibiotics, China annually produces approximately 210 000 tons of antibiotics.^3^ However, TC is metabolized slowly in organisms and prone to accumulation.^4^ It can also be passively absorbed through the food chain, posing risks to human health, including hepatorenal toxicity, gastrointestinal disturbances, and impaired skeletal and dental development.^5,6^ The ecological and public health concerns arising from TC overuse have garnered significant attention.^7^

Current detection techniques for TC, such as high-performance liquid chromatography (HPLC), liquid chromatography-mass spectrometry (LC-MS), electrochemical analysis, capillary electrophoresis (CE), and enzyme-linked immunosorbent assay (ELISA), are limited by operational complexity, high cost, and inability to facilitate rapid on-site analysis.^7–10^ Consequently, there is an urgent need to develop rapid, sensitive, selective, and cost-effective detection methods.^11^ Although fluorescence-based approaches have shown promise for TC detection, the synthesis of conventional fluorescent probes often entails high energy consumption and cost.^12–14^

Carbon quantum dots (CQDs), as an emerging class of fluorescent nanomaterials, have attracted considerable interest in biomedical imaging, photocatalysis, sensing, and analytical detection due to their exceptional optical properties, low cost, eco-friendliness, high stability, and good biocompatibility.^15–19^ Synthesis strategies for CQDs are broadly classified into top-down and bottom-up approaches, including arc discharge, electrochemical oxidation, laser ablation, hydrothermal treatment, and microwave-assisted synthesis.^19–23^ Among these, the hydrothermal method is commonly used but often requires prolonged reaction times and high energy input. In contrast, microwave-assisted synthesis offers advantages such as rapid and uniform heating, reduced energy consumption, shorter reaction duration, and operational simplicity, making it an increasingly attractive option.^1,24^

The precursor materials for CQD synthesis are highly diverse, ranging from pure carbon sources (*e.g.*, graphite, carbon nanotubes)^25,26^ to organic molecules (*e.g.*, citric acid, urea, amino acids),^27,28^ and more recently, to abundant, low-cost, and renewable waste biomass.^29,30^ The use of waste biomass not only enables high-value resource recovery but also provides a sustainable and green pathway for CQD production.^31^ Various biomass wastes, including barley bran,^32^ banana peel,^33^ plant leaves,^34^ and crab shell,^35^ have been successfully utilized to synthesize fluorescent CQDs.

Spent coffee grounds (SCGs) constitute a major organic waste stream generated from coffee processing.^36^ With rising global coffee consumption, SCG production has increased substantially.^37^ SCGs are rich in polysaccharides (*e.g.*, cellulose, hemicellulose, lignin), polyphenols, lipids, and minerals, enabling their valorization in food, agriculture, and industrial applications.^38,39^ Current SCG utilization pathways include use as biomass fuel,^40,41^ sunscreen ingredients,^42^ carriers for biomolecules, and soil compost.^43^ Moreover, as a carbon-rich waste, SCGs can be converted into carbon dots (CDs) with uniform morphology, good water dispersibility, and tunable fluorescence *via* green synthesis methods such as hydrothermal treatment and ball milling.^38,44^ These CDs show great potential as function nanomaterials for environmental monitoring and as nanofillers in biopolymer-based active packaging films. For instance, Nazar *et al.* used SCG-derived carbon dots for the detection Fe^3+^, Pb^2+^, and Cr^3+^;^45^ Zhu *et al.* developed a simple hydrothermal approach to synthesize CQDs from SCGs for ascorbic acid sensing;^46^ and Gao *et al.* applied SCG-based CDs into food packaging films to inhibit microbial spoilage.^47^ However, the application of SCG-derived CQDs for the sensitive and selective detection of tetracycline antibiotics has not yet been reported.

In this study, we utilized spent coffee grounds—a renewable, low-cost, and carbon/nitrogen-rich biomass—as a precursor to synthesize high-performance coffee ground-derived carbon quantum dots (B-CQDs) *via* a green and efficient microwave-assisted method. The resulting B-CQDs were employed as a novel fluorescent probe for the highly selective and sensitive detection of TC. The established method provides a sustainable and eco-friendly analytical platform for the rapid, low-cost, and real-time monitoring of TC residues in water, holding significant promise for applications in environmental and food safety.

## 2. Materials and methods

### 2.1 Reagents and materials

Spent coffee grounds (SCGs) were collected from a Luckin Coffee outlet on campus. Tetracycline hydrochloride (TC), KCl, NaCl, CaCl_2_, ZnCl_2_, MgCl_2_, NiCl_2_·6H_2_O, CuSO_4_·5H_2_O, FeSO_4_·7H_2_O, FeCl_3_·6H_2_O, CoCl_2_·6H_2_O, Na_2_CO_3_, NaH_2_PO_4_, NaNO_3_, NaOH, and quinine sulfate were purchased from Tianjin Synthgene Medical Technology Co., Ltd. NaNO_3_ and sulfuric acid were supplied by Tianjin Jiangtian Chemical Technology Co., Ltd. Gentamicin sulfate (GEN), streptomycin sulfate (STR), spectinomycin (SPE), and kanamycin sulfate (KAN) were obtained from Beijing Solarbio Technology Co., Ltd. Phosphoric acid, boric acid, and acetic acid were procured from Kemate Chemical Technology Co., Ltd. Microporous membranes (0.22 µm) and dialysis bags (MWCO 500 Da) were acquired from Tianjin Leviathan Technology Co., Ltd. All chemicals were of analytical grade, and all solutions were prepared using double-distilled water.

### 2.2 Synthesis and purification of B-CQDs

**Fig. 1:**
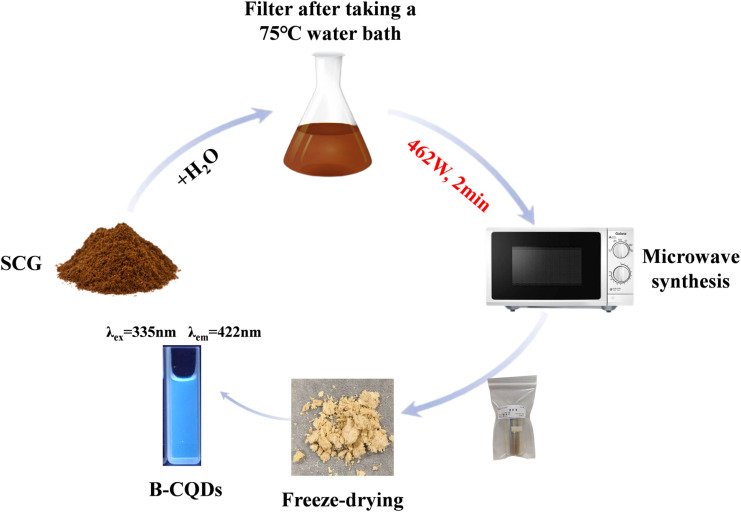
Schematic illustration of the synthesis procedure for B-CQDs.

The collected SCGs were thoroughly washed with ultrapure water and dried in an oven at 60 °C. Subsequently, 0.5 g of dried SCGs were mixed with 20 mL of deionized water in a 50 mL conical flask. The mixture was sonicated for 10 min and then incubated in a water bath at 75 °C for 20 min. After filtration, the solution was subjected to microwave irradiation in a domestic microwave oven at 462 W (medium power) for 2 min. The resulting product was centrifuged at 6000 rpm for 15 min. The supernatant was filtered through a 0.22 µm membrane to remove insoluble particles and macromolecular impurities. The filtrate was dialyzed using a dialysis membrane (MWCO 500 Da) against ultrapure water for 24 h, with the water changed every 12 h. Finally, the purified solution was freeze-dried to obtain a light-yellow fluffy powder of B-CQDs, which was stored at 4 °C for further use. The synthesis procedure is schematically illustrated in Fig. 1.

### 2.3 Characterization of B-CQDs

The morphology of the synthesized B-CQDs was characterized by transmission electron microscopy (TEM, JEOL JEM-2100, Japan). X-ray diffraction (XRD) patterns were recorded on a diffractometer (Rigaku Rigaku-2038, Japan) over a 2*θ* range of 5° to 80°. Fourier transform infrared (FT-IR) spectra were acquired using a spectrometer (Bruker INVENIO-S, Germany) in the wavenumber range of 4000–400 cm^−1^. Elemental composition and chemical states were analyzed by X-ray photoelectron spectroscopy (XPS, Thermo Scientific K-Alpha, USA). Fluorescence excitation and emission spectra were measured on a fluorescence spectrophotometer (F-2500, Hitachi, Japan). UV-Vis absorption spectra were obtained using a spectrophotometer (CARY 100, Varian, USA) in the wavelength range of 200–600 nm. Fluorescence lifetimes were determined by a steady-state/transient fluorescence spectrometer (FLS 1000, UK Edinburgh).

### 2.4 Fluorescence quantum yield calculation

The fluorescence quantum yield (QY) of B-CQDs was determined using quinine sulfate (dissolved in 0.1 M H_2_SO_4_, QY = 0.54 at 360 nm excitation) as a reference standard, according to a previously reported method.^48^ The QY was calculated using the following equation:^45,49^
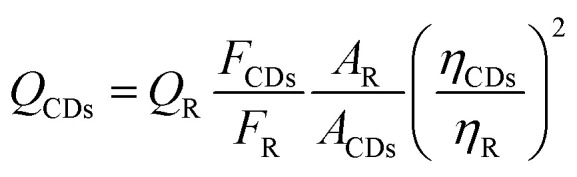
where *Q* is the quantum yield, *F* is the integrated fluorescence intensity, *A* is the absorbance at the excitation wavelength, and *η* is the refractive index of the solvent. The subscripts R and CDs refer to the reference (quinine sulfate) and B-CQDs, respectively. The absorbance of all samples was kept below 0.05 to avoid inner filter effects.

### 2.5 Stability of B-CQDs

The stability of B-CQDs was evaluated under various environmental conditions. For salt tolerance analysis, B-CQDs solution (500 µL) was mixed with NaCl solution (1000 µL) to achieve final concentrations of 0–500 mmol L^−1^. After 5 min equilibration at room temperature, fluorescence intensity was measured at an excitation wavelength of 335 nm. For photostability analysis, the B-CQDs solution was exposed to continuous UV light (365 nm), and fluorescence intensity was recorded every 10 min over 60 min. For pH stability analysis, fluorescence intensity was measured in buffer solutions with pH values ranging from 2.18 to 11.92. For temperature-dependent quenching, B-CQDs solution (3 mL) was mixed with tetracycline solution (100 mmol L^−1^) to achieve final concentrations of 20–60 µmol L^−1^, then treated the B-CQDs solution and B-CQDs-tetracycline solution at 293 K, 313 K, and 333 K for 5 min, then test their fluorescence intensity and calculate the Stern–Volmer constant (*K*_SV_):
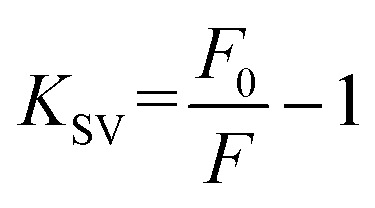
where *F*_0_ represent fluorescence intensities in the absence and *F* represent fluorescence intensities in the presence of TC.

### 2.6 Detection of tetracycline

For tetracycline detection, 750 µL of B-CQDs solution (300 mg L^−1^) was mixed with 750 µL of TC solution at varying concentrations (0–140 µmol L^−1^). After 1 min of reaction at room temperature, fluorescence intensity was measured at an excitation wavelength of 335 nm.

### 2.7 Selectivity and interference experiments

Selectivity was assessed against metal ions (K^+^, Na^+^, Ca^2+^, Mg^2+^, Zn^2+^, Ni^2+^, Co^2+^, Cu^2+^, Fe^2+^, Fe^3+^), common anions (Cl^−^, NO_3_^−^, SO_4_^2−^, CO_3_^2−^, PO_4_^3−^), and antibiotics (GEN, STR, SPE, KAN). The concentration of each potential interferent was 160 µmol L^−1^, while TC concentration was set at 80 µmol L^−1^. In selectivity tests, interferents were added individually to the B-CQDs solution instead of TC. In interference tests, both the interferent and TC were present simultaneously. Fluorescence spectra were recorded in each case.

### 2.8 Real sample analysis

Water samples including pond water (campus), tap water (laboratory), and wastewater (swine farm, Tianjin) were centrifuged at 6000 rpm for 10 min and filtered through a 0.22 µm membrane. Tetracycline was spiked into the pre-treated samples at final concentrations of 10 and 40 µmol L^−1^. Then, 750 µL of each spiked sample was mixed with 750 µL of B-CQDs solution, and fluorescence intensity was measured. All experiments were performed in triplicate.

### 2.9 Greenness evaluation metric for analytical methods (GEMAM)

The greenness of the proposed method was evaluated using GEMAM, a multi-criteria tool based on the 12 principles of green analytical chemistry and 10 factors of green sample preparation.^50^ The assessment was performed with the publicly available GEMAM software (https://gitee.com/xtDLUT/Gemam), using default weightings: reagents (25%), waste (25%), method (15%), instrument (15%), sample (10%), and operator (10%). The tool generates a visual pictogram reflecting the method's environmental performance across six dimensions.

## 3. Results and discussion

### 3.1 Morphology, structure, and formation mechanism of B-CQDs

The synthesis conditions for B-CQDs were optimized using a single-factor experiment (Fig. S1). The results indicated that the highest fluorescence intensity of the prepared B-CQDs was achieved under the following conditions: microwave power of 462 W (Fig. S1A), irradiation time of 2 min (Fig. S1B), and coffee ground mass of 0.5 g (Fig. S1C). Microwave irradiation enables rapid and uniform bulk heating through intermolecular friction, significantly accelerating the carbonization and passivation processes of components such as carbohydrates and proteins present in coffee grounds.^51^ However, excessive irradiation time or power may lead to overcarbonization of the carbon core or degradation of surface functional groups, thereby reducing the fluorescence quantum yield. This is consistent with the observed trend of initial increase followed by a decrease in fluorescence intensity shown in Fig. S1.

**Fig. 2:**
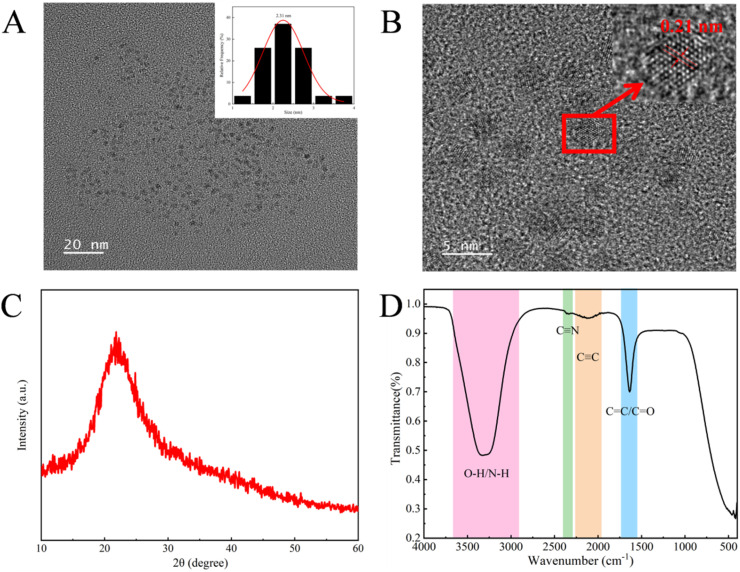
Structural characterization of B-CQDs: (A) TEM image (inset: particle size distribution histogram); (B) HRTEM image; (C) XRD pattern; (D) FT-IR spectrum.

TEM and HRTEM results (Fig. 2A and B) confirmed the successful synthesis of carbon quantum dots with uniform size and good crystallinity. As shown in Fig. 2A, the B-CQDs exhibited a near-spherical morphology, good dispersibility, and a particle size distribution ranging from 1.25 to 3.75 nm, with an average diameter of 2.31 nm. The HRTEM image (Fig. 2B) revealed distinct lattice fringes with an interplanar spacing of approximately 0.21 nm, corresponding to the (100) crystal plane of graphitic carbon, which is consistent with previously reported findings (Li *et al.*, 2014; Xu *et al.*, 2020). This indicates that despite the rapid microwave treatment, the process still induced the formation of a core structure containing sp^2^-hybridized carbon domains, which serves as the structural foundation for the favorable fluorescence properties of the B-CQDs.^52,53^

The XRD pattern (Fig. 2C) displayed a broad diffraction peak centered at approximately 21.9 (2*θ*), suggesting that the B-CQDs have lower crystallinity,^54^ which is mainly attributed to their relatively mild synthesis conditions and the complex chemical composition of the precursor. These factors collectively hinder the formation of highly ordered graphitic crystals.

FT-IR spectroscopy (Fig. 2D) revealed broad absorption peaks at 3322 cm^−1^ and 2111 cm^−1^, attributed to O–H/N–H stretching vibrations and C≡C stretching, respectively. The absorption peak at 1624 cm^−1^ corresponds to C=C/C=O stretching vibrations, while the weak peak near 2349 cm^−1^ may originate from C≡N bonds. The presence of these functional groups indicates that the B-CQDs' surface is rich in hydrophilic groups such as hydroxyl and carboxyl groups, endowing them with good water dispersibility.^55,56^

**Fig. 3:**
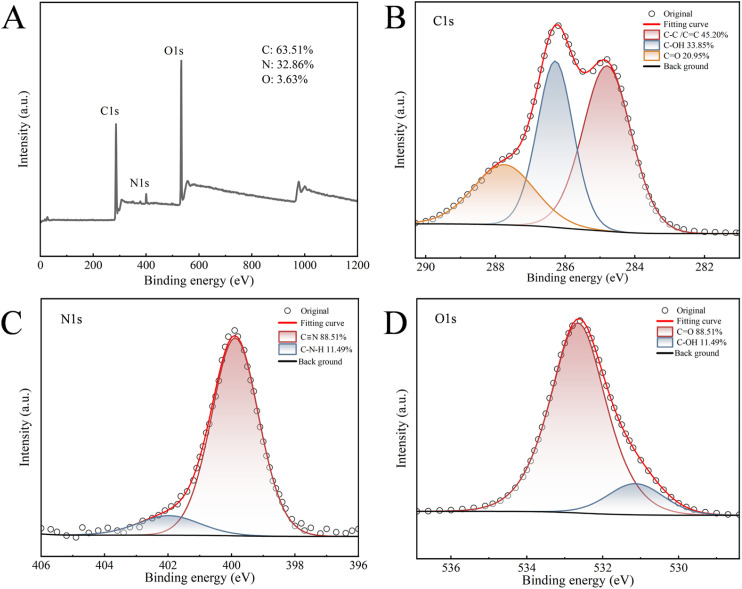
XPS analysis of B-CQDs: (A) full survey spectrum; high-resolution spectra of (B) C 1s, (C) O 1s, and (D) N 1s.

The XPS survey spectrum (Fig. 3A) confirmed that B-CQDs are primarily composed of three elements: C (63.51%), O (32.86%), and N (3.63%). The high-resolution C 1s spectrum (Fig. 3B) could be deconvoluted into three peaks corresponding to C–C/C=C (284.8 eV), C–OH/C–N (286.29 eV), and C=O (287.76 eV). The O 1s spectrum (Fig. 3C) was fitted with components for C=O (531.14 eV) and C–OH (532.65 eV). The N 1s spectrum (Fig. 3D) indicated the presence of nitrogen-containing functional groups such as C–N–C (399.89 eV) and N–H (401.97 eV). The XPS results further corroborate the abundance of oxygen- and nitrogen-containing functional groups on the B-CQDs' surface, contributing to their good aqueous dispersion stability, which is consistent with the FT-IR analysis.

Collectively, FT-IR and XPS analyses revealed that the B-CQDs' surface is rich in hydroxyl, carboxyl, carbonyl, and nitrogen-containing functional groups. Their presence can be attributed to the inherently diverse chemical composition of the spent coffee grounds (*e.g.*, cellulose, hemicellulose, proteins) and potentially to their formation during the microwave-assisted hydrolysis and oxidation process. These hydrophilic groups are key to the excellent aqueous dispersion stability of the B-CQDs. More importantly, they not only contribute to the surface-state fluorescence but also provide potential binding sites for subsequent interactions with tetracycline molecules, which is crucial for their performance as a sensing probe.

### 3.2 Optical properties and stability of B-CQDs

**Fig. 4:**
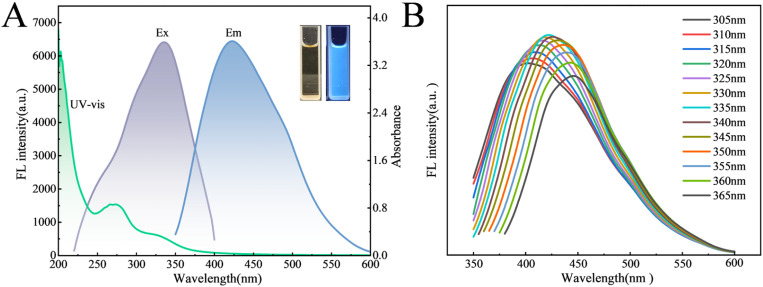
Optical properties of B-CQDs: (A) UV-Vis absorption spectrum (green), fluorescence excitation spectrum (purple), and emission spectrum (blue); inset: photographs of B-CQDs solution under daylight (left) and 365 nm UV light (right). (B) Fluorescence emission spectra under different excitation wavelengths.

The optical properties of B-CQDs were further characterized by fluorescence and UV-Vis absorption spectroscopy. Fig. 4A showed that B-CQDs emits blue fluorescence under UV light and appears as a clear, light-yellow solution under daylight. The UV-Vis absorption spectrum (Fig. 4A) exhibited a distinct absorption peak between 300 and 350 nm, attributed to π–π* electronic transitions, indicating the formation of a conjugated skeleton,^57^ consistent with many previous reports.^58–60^ Fluorescence spectra (Fig. 4B) reveal that the emission peak of B-CQDs gradually red-shifts from 403 nm to 446 nm as the excitation wavelength increases from 305 nm to 365 nm, demonstrating excitation-dependent emission behavior. This phenomenon is commonly ascribed to the presence of sp^2^ carbon domains of varying sizes and diverse surface emission states in the carbon dots.^61^

When the excitation wavelength changes, different emissive centers at various energy levels are selectively excited, resulting in the observed red-shift and variation in fluorescence intensity. The maximum emission intensity was achieved at 422 nm under 335 nm excitation. Using quinine sulfate as a reference, the fluorescence quantum yield (QY) of B-CQDs in water was calculated to be 11.2%, which is higher than that of many previously reported coffee ground-derived carbon dots (Table S1), providing an advantage for sensing applications. We speculate that this enhanced QY benefits from the well-defined carbon core efficiently formed *via* microwave irradiation and the self-doping effect of nitrogen naturally present in coffee grounds. The incorporation of nitrogen can create new energy levels within the carbon framework, thereby improving the radiative recombination efficiency.^62,63^

**Fig. 5:**
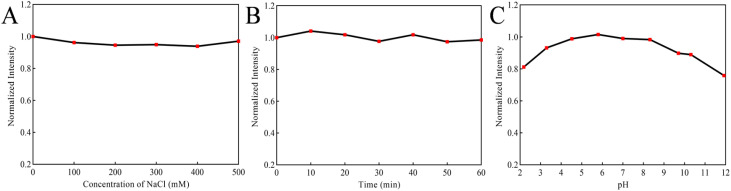
Stability evaluation of B-CQDs: (A) Effect of ionic strength (NaCl concentration: 0–500 mM) on fluorescence intensity; (B) photostability under continuous 365 nm UV irradiation; (C) pH dependence of fluorescence intensity (pH range: 2.18–11.92).

The stability of B-CQDs was evaluated by examining the effects of salt concentration, UV irradiation time, and pH on their fluorescence intensity (excited at 335 nm).^64^ The salt tolerance was investigated by measuring the fluorescence intensity of B-CQDs solutions with NaCl concentrations of 0, 100, 200, 300, 400, and 500 mmol L^−1^. As shown in Fig. 5A, the fluorescence intensity of B-CQDs was not significantly affected across the tested NaCl concentrations, indicating excellent tolerance to high salinity conditions. The photostability was assessed by continuously irradiating the B-CQDs solution with UV light for 60 minutes and measuring the fluorescence intensity every 10 minutes. After 60 minutes of irradiation, the fluorescence intensity remained above 95% of its initial value (Fig. 5B), demonstrating strong resistance to photobleaching. Furthermore, the pH stability tests indicated that the fluorescence of B-CQDs remained stable in weakly acidic to weakly alkaline environments (Fig. 5C). The fluorescence quenching observed under strongly acidic or alkaline conditions can be attributed to the protonation/deprotonation of surface carboxyl or amino groups.^60^ This process alters the surface charge state and disrupts the intramolecular charge transfer (ICT), thereby activating non-radiative transition pathways.^65^ The measured pH of the B-CQDs aqueous solution was 6.67, at which the fluorescence intensity was stable. Therefore, no pH adjustment was necessary for subsequent measurements. These findings indicated that the prepared B-CQDs possess excellent stability, making them promising fluorescent probes for practical applications.

### 3.3 Specific detection of TC by B-CQDs

Prior to TC detection, we first optimize conditions such as B-CQDs concentration, pH, and detection time. As shown in Fig. S3A, the fluorescence quenching rate (*Q*_E_) of the probe initially increased and then gradually decreased as the B-CQDs concentration increased from 50 mg L^−1^ to 500 mg L^−1^, reaching a maximum at 150 mg L^−1^. The *Q*_E_ remained relatively stable in the pH range of 2.09–8.32 but decreased gradually at higher pH values (Fig. S3B), probably due to deprotonation of surface groups on B-CQDs and increased repulsion with TC.^64,66^ The pH of the B-CQDs aqueous solution was 6.67, where the *Q*_E_ was stable (Fig. S3C). Therefore, a concentration of 150 mg L^−1^ and a reaction time of 1 minute were selected as optimal conditions for subsequent experiments without pH adjustment.

**Fig. 6:**
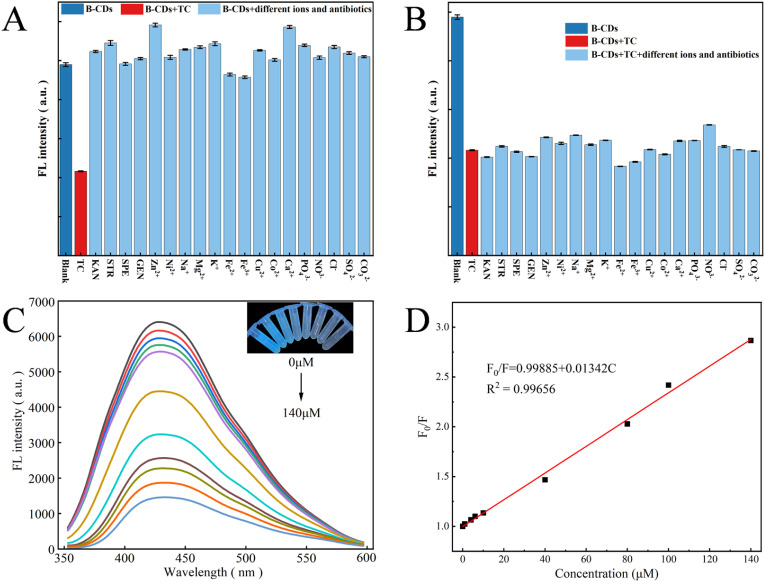
Sensing performance of B-CQDs for tetracycline detection: (A) selectivity toward various interferents; (B) interference resistance in the presence of TC and other substances; (C) fluorescence emission spectra with increasing TC concentrations (0–140 µmol L^−1^); (D) linear relationship between *F*_0_/*F* and TC concentration. All data represent mean values from triplicate measurements.

To evaluate the selectivity of B-CQDs for TC detection, changes in fluorescence intensity were measured in the presence of various ions (K^+^, Zn^2+^, Ni^2+^, Na^+^, Mg^2+^, Ca^2+^, Co^2+^, Cu^2+^, Fe^2+^, Fe^3+^) and antibiotics (GEN, STR, SPE, KAN) as well as common anions (Cl^−^, SO_4_^2−^, NO_3_^−^, CO_3_^2−^, PO_4_^3−^). The results showed that only TC caused significant fluorescence quenching of B-CQDs, demonstrating high selectivity for TC recognition (Fig. 6A). To assess potential interference in practical detection, fluorescence intensity was measured in the presence of TC along with other ions and antibiotics. As shown in Fig. 6B, the fluorescence of B-CQDs could still be quenched by TC even in the presence of various interferents, indicating excellent anti-interference capability. In summary, the high selectivity and anti-interference performance of B-CQDs make them suitable as fluorescent probes for detecting TC in environmental water samples.

The fluorescence intensity of B-CQDs (*λ*_ex_ = 335 nm) gradually decreased as the TC concentration increased from 0 to 140 µmol L^−1^ (Fig. 6C). A good linear correlation was observed between *F*_0_/*F* and TC concentration (where *F* represents the fluorescence intensity of CQDs after adding TC, and *F*_0_ is the blank value). As shown in Fig. 6D, the linear regression equation was determined as *F*_0_/*F* = 0.01342*C* + 0.99885, with a correlation coefficient (*R*^2^) of 0.99656. The limit of detection (LOD), calculated as LOD = 3*δ*/*k* (where *δ* is the standard deviation of the fluorescence intensity of blank B-CQDs solution (*n* = 10), and *k* is the slope of the linear equation), was 0.36 µmol L^−1^.

The analytical performance of the B-CQDs probe was compared with other fluorescence-based sensors derived from biomass, as summarized in Table S2. The developed sensor demonstrates a highly competitive profile. Its detection limit (0.36 µmol L^−1^) is comparable to or lower than that of many existing probes, such as those derived from red beet pigment (RBP-CDs, 0.36 µmol L^−1^)^67^ and wild lemon leaves (LLCDs, 0.42 µmol L^−1^).^68^ More notably, it offers a significantly wider linear range (0–140 µmol L^−1^) than most counterparts, encompassing both trace-level and high-concentration scenarios, which is a distinct practical advantage. Crucially, the excellent recovery rates (98.8–105.5%) and low RSDs (<3.53%) achieved in the analysis of various real water samples (Table 1) confirm its superior anti-interference capability and reliability in complex environmental matrices. This combination of a low LOD, a wide linear range, and robust performance in real samples, coupled with the green and cost-effective synthesis from waste biomass, underscores the strong potential of the B-CQDs for practical TC monitoring.

**Table 1:** Determination of tetracycline (TC) in different real water samples

Sample	Added (µmol L^−1^)	Detected (µmol L^−1^)	Recovery rate (%)	RSD% (*n* = 3)
Tap water	10	9.88	98.8	2.27
	40	40.5	101.1	1.18
Lake water	10	10.23	102.3	2.85
	40	42.19	105.5	3.53
Swine farm wastewater	10	10.18	101.8	2.78
	40	41.45	103.6	3.42

### 3.4 Application in real samples

To validate the applicability of B-CQDs in real water samples, lake water from the campus, tap water, and wastewater from a swine farm in Tianjin were selected for analysis. The TC concentration in these actual samples was determined using the standard addition method. As shown in Table 1, the recovery rates of TC in the spiked water samples ranged from 98.8% to 105.5%, with the relative standard deviation (RSD) not exceeding 3.53% (*n* = 3). The results demonstrate that the proposed method offers good accuracy and holds considerable promise for the detection of TC in environmental water samples.

### 3.5 Mechanism of detecting TC

**Fig. 7:**
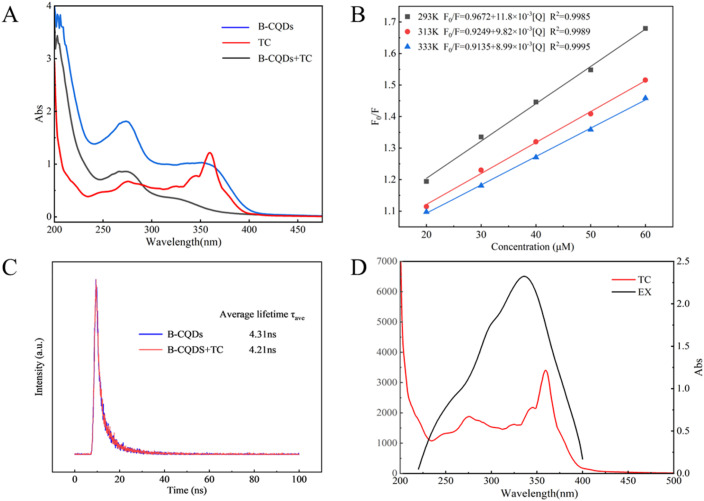
Fluorescence quenching mechanism: (A) UV-Vis absorption spectra of B-CQDs, TC, and their mixture; (B) fluorescence lifetime of R-CDs in the absence and presence of TC. (C) Stern–Volmer plots of *F*_0_/*F versus* TC concentration at different temperatures. (D) Overlap between the UV absorption spectrum of TC and the excitation spectrum of B-CQDs.

To further investigate the potential sensing mechanism of B-CQDs for TC, the UV-Vis absorption spectra of solutions containing B-CQDs, TC, and B-CQDs + TC were compared. As shown in Fig. 7A, characteristic absorption peaks of TC were observed at 276 nm, 346 nm, and 360 nm. Significant spectral changes were observed upon the addition of TC to the B-CQDs solution, which is likely attributed to static quenching resulting from the interaction between TC and B-CQDs.^69^

To verify this, fluorescence lifetime and temperature-dependent quenching studies were conducted. As illustrated in Fig. 7B, the *K*_SV_ values at 293 K, 313 K, and 333 K were measured as 11.8 × 10^3^ L mol^−1^, 9.82 × 10^3^ L mol^−1^ and 8.99 × 10^3^ L mol^−1^, respectively. The results shown that *K*_SV_ decreases with the increase of temperature, which is consistent with the characteristics of static quenching. Furthermore, the near-invariance of the B-CQDs' fluorescence lifetime upon TC addition (Fig. 7C) conclusively rules out dynamic quenching and supports a static quenching pathway.^70,71^

Moreover, a noticeable spectral overlap region was observed between the absorption spectrum of TC and the maximum excitation peak of B-CQDs (Fig. 7D), indicating that an inner filter effect (IFE) may also contribute to the fluorescence quenching of B-CQDs by TC.^72,73^ In conclusion, both static quenching and IFE contribute to the fluorescence quenching of B-CQDs induced by TC. The elucidation of these two underlying mechanisms in this study reinforces the potential of B-CQDs for selective and sensitive detection of tetracycline, enabling minimal interference even in complex environments and demonstrating promising application prospects.

### 3.6 Assessment of method greenness using the GEMAM

The growing emphasis on sustainable analytical practices has led to the development of various green metric tools. For instance, Jalal *et al.* evaluated their method using the Analytical GREEnness (AGREE) and Blue Applicability Grade Index (BAGI),^74^ while Elshenawy *et al.* applied the Green Analytical Procedure Index (GAPI) along with AGREE.^75^ Both studies successfully demonstrated the favorable environmental profiles of their respective methods.

Compared to these established tools, the Greenness Evaluation Metric for Analytical Methods (GEMAM) adopted in this study offers a more integrated and comprehensive assessment framework. GEMAM combines the 12 principles of Green Analytical Chemistry (GAC) with the 10 key factors of green sample preparation into a unified system. This integration allows a single evaluation to yield both a quantitative score and an intuitive visual pictogram, eliminating the need for multiple separate assessments. Furthermore, GEMAM covers a broader range of dimensions, including sample and reagent consumption, instrumental requirements, waste generation, and operator safety.

**Fig. 8:**
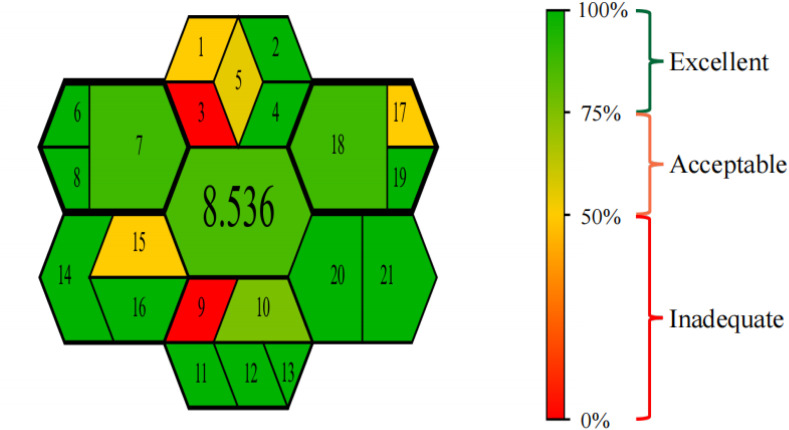
GEMAM assessment results: (Left) overall greenness profile; (Right) corresponding color scale.

The GEMAM pictogram and overall score for the proposed method are shown in Fig. 8. The method achieved a high greenness score of 8.536 out of 10, reflecting outstanding environmental friendliness. This result is primarily attributed to the valorization of waste biomass, effective avoidance of hazardous chemicals, and enhanced operational safety—all of which align closely with the principles of green chemistry and the circular economy. Comprehensive analysis confirms that the proposed method exhibits superior eco-compatibility, greenness, and operational safety, positioning it as an environmentally sustainable analytical strategy.

Furthermore, it is pertinent to discuss the sustainability and practicality of the nanomaterial synthesis itself. Although this study employed a microwave-assisted route due to its remarkable rapidity (a few minutes), high energy efficiency, and operational simplicity, we acknowledge its limitations for large-scale production owing to the restricted volume of common laboratory microwave reactors. In contrast, the conventional hydrothermal method, despite requiring considerably longer reaction times (typically several hours), is inherently more amenable to scaling up by utilizing larger autoclaves. The microwave approach is ideally suited for the rapid, on-demand synthesis of small batches, which presents a significant advantage for sensor development and laboratory research. Future work could explore continuous-flow microwave reactors as a potential pathway to overcome this scale-up limitation while retaining the benefits of rapid heating.^76–78^

## 4. Conclusion

This study developed a simple, green, and reproducible one-step microwave-assisted method to synthesize B-CQDs using spent coffee grounds as the raw material. The results demonstrated that the as-prepared B-CQDs possess uniform particle size (average diameter ≈ 2.31 nm), good water dispersibility, excellent fluorescence stability, and a high QY of 11.2%. Moreover, the B-CQDs can serve as an efficient and sensitive fluorescent probe for the specific detection of TC. The detection based on fluorescence quenching exhibited a relatively low LOD (0.36 µmol L^−1^) and a good linear relationship with TC concentration. The quenching mechanism was primarily attributed to IFE and static quenching. In the analysis of real environmental water samples, the method delivered satisfactory statistical results, confirming its reliability and practicality. In summary, this work not only offers a new approach for the resource utilization of waste biomass, but also establishes a fluorescence sensing platform with strong practical potential for monitoring TC in environmental water systems.

## Author contributions

Haochen Shen: investigation, methodology, formal analysis, visualization, writing – original draft. Ying Chu: investigation, methodology, data curation, validation, writing – original draft. Ziyi Liu: resources, data curation. Chuhan Zhang and Yang Yu: sample processing, investigation, visualization. Shaohui Yang: funding acquisition, conceptualization, supervision, validation, writing – review & editing.

## Conflicts of interest

The authors have declared that there is no conflict interest.

## Supplementary Material

RA-016-D5RA07629C-s001

## Data Availability

All the data that support the findings of this study are included in the manuscript and supplementary information (SI). Supplementary information contains the synthesis of carbon quantum dots and the detection of their indicators, comparisons with existing literature, and the supplementation of the results. See DOI: https://doi.org/10.1039/d5ra07629c.
