# Supplementary material for: Highly selective sensing of tetracycline by fluorescent carbon dots derived from spent coffee grounds via a green microwave route
Source: RSC Adv. 2026 Jan 1;16(1):797–807. doi: 10.1039/d5ra07629c (PMC12757101; doi:10.1039/d5ra07629c)

Supplementary Information (SI) for RSC Advances.
This journal is © The Royal Society of Chemistry 2025

Highly selective sensing of tetracycline by fluorescent carbon dots derived from spent coffee grounds via a green microwave route

Haochen Shen[1, 2], Ying Chu[1], Ziyi Liu[1], Chuhan Zhang[1], Yang Yu[1], Shaohui Yang[1]*

[1]School of Environmental Science and Engineering, Tianjin University, Tianjin 300354, China

[2]Teda Greening Science and Technology Group Co., LTD., Tianjin 300457, China

*Corresponding author. E-mail: shaohuiyang77@tju.edu.cn

Supplemental Method 1. Optimization of carbon quantum dots synthesis conditions

Initially, with the amount of spent coffee grounds fixed at 1.0 g and the heating time set at 4 minutes, carbon dots were synthesized under different microwave power levels (119, 280, 462, 595, and 700 W). The fluorescence intensity of the resulting carbon dots was measured at an excitation wavelength of 335 nm. Following the identification of the optimal microwave power, the amount of spent coffee grounds was maintained at 1.0 g, and the heating time was varied (2, 4, 6, and 8 minutes) to synthesize carbon dots, whose fluorescence intensity was subsequently determined. Finally, under the optimal microwave power and heating time conditions, carbon dots were synthesized using different amounts of spent coffee grounds (0.5, 1.0, 1.5, 2.0, and 2.5 g), and their fluorescence intensity was measured.

Supplemental Method 2. Optimization of tetracycline (TC) detection conditions

To ensure the sensitivity and optimal performance of the coffee ground-derived carbon quantum dots (B-CQDs) in practical TC detection, several key parameters were optimized, including the concentration of B-CQDs, pH, and detection time. First, different concentrations of B-CQDs (50, 100, 150, 200, 300, 400, and 500 mg/L) were used to detect TC (100 μM) in a total reaction volume of 2 mL. The fluorescence spectra were recorded under an excitation wavelength of 335 nm. The influence of B-CQDs concentration on detection performance was evaluated based on the fluorescence quenching efficiency (Q_E), calculated using the following formula:

$$Q_E = \frac{F_0 - F}{F_0} \times 100\%$$

Where F_0 and F represent the fluorescence intensity in the absence and presence of TC, respectively. Next, the optimal pH was determined using BR buffer solutions at various pH values (2.18, 3.29, 4.52, 5.80, 7.00, 8.32, 9.70, 10.31, and 11.92). The reaction mixture contained B-CQDs at a concentration of 150 mg/L, TC at 100 μM, and 1.5 mL of BR buffer, with a total volume of 2 mL. Fluorescence spectra were acquired, and the Q_E was used to identify the best pH condition. Finally, the detection time was optimized

by monitoring the fluorescence spectra of the mixture over a time range of 0 to 30 minutes. The Q_E values were calculated at different time points to determine the optimal detection time.

Supplemental Table 1. Comparison of quantum yields of B-CQDs in this study with other spent coffee grounds（SCG）derived carbon dots in the literature.

Carbon dots	Quantum yield（%）	Reference
CQDs	6	[1]
CDs	2.9-5.8	[2]
C-dots	5.5	[3]
C-dots	3.8	[4]
CDs	5	[5]
Fe-CQDs	4.18	[6]
B-CQDs	11.2	This work

[1] Nazar, M., et al., (2024). Microwave synthesis of carbon quantum dots from arabica coffee ground for fluorescence detection of Fe^{3+}, Pb^{2+}, and Cr^{3+}. ACS Omega 9 (18), 20571–20581.

[2] Costa, A. I.; Barata, P. D.; Moraes, B.; Prata, J. V. (2022) Carbon dots from coffee grounds: synthesis, characterization and detection of noxious nitroanilines. Chemosensors, 10(3), 113

[3] Crista, D. M. A., El Mragui, A., Algarra, M., Esteves da Silva, J. C. G., Luque, R., & Pinto da Silva, L. (2020). Turning spent coffee grounds into sustainable precursors for the fabrication of carbon dots. Nanomaterials,10(6), 1209.

[4] Chengkun Jiang, Hao Wu, Xiaojie Song, Xiaojun Ma, Jihui Wang, Mingqian Tan, (2014) Presence of photoluminescent carbon dots in Nescafe® original instant coffee: Applications to bioimaging, Talanta, 127, 68-74, Hsu, P.C.; Shih, Z.Y.; Lee, C.H.; Chang, H.T. Synthesis and analytical applications of photoluminescent carbon nanodots. Green Chem. 2012, 14, 917–920.

[5] Yuyan Zhu, Xiang Deng, Jiaming Chen, Zhiyuan Hu, Fengshou Wu, (2023) Coffee grounds-derived carbon quantum dots as peroxidase mimetics for colorimetric and fluorometric detection of ascorbic acid, Food Chemistry, 429, 136957

Supplemental Table 2. Comparison of fluorometric detection of TC

Probes	Material	Linear range (μM)	LOD (μM)	Reference
RBP-CDs	Red beet pigment	0.5-90	0.36	[7]
LLCDs	Wild lemon leaves	0-27	0.42	[8]
E-CQDs	*Eucheuma denticulatum*	20-100	0.47	[9]
CJ-CDs	Carrot juice	4-15.5	1.33	[10]
B-CQDs	Spent coffee grounds	0–140	0.36	This work

[6] Y. Cao. (2022) Fluorescent detection of tetracycline in foods based on carbon dots derived from natural red beet pigment, LWT, 157, 113100

[7] Venugopalan P, Vidya N. (2023) Microwave-assisted green synthesis of carbon dots derived from wild lemon (*Citrus pennivesiculata*) leaves as a fluorescent probe for tetracycline sensing in water. Spectrochim Acta A Mol Biomol Spectrosc. 286, 122024

[8] Li, W., Luo, K., Lv, M.et al. (2021) Carbon quantum dots derived from marine *Eucheuma denticulatum* for the efficient and sensitive detection of tetracycline. J Nanopart Res 26, 70

[9] M. Mohammadnejad, R. Alekasir, (2024) Sensitive and rapid determination of tetracycline antibiotic by carrot juice-derived carbon dots as a fluorescent probe, Luminescence 39 (7) e4828.

Supplemental Fig. 1 Normalized fluorescence intensity of B-CQDs synthesized at different microwave powers (A), different reaction times (B), and different dosages of spent coffee grounds (C).

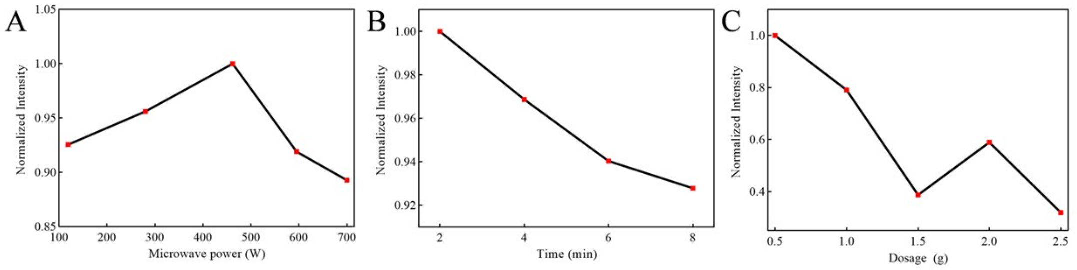

Supplemental Fig. 2 Quantum yield of B-CQDs in different solvents, WAT: water， MET: methanol, ETH: ethanol, ACE: acetone.

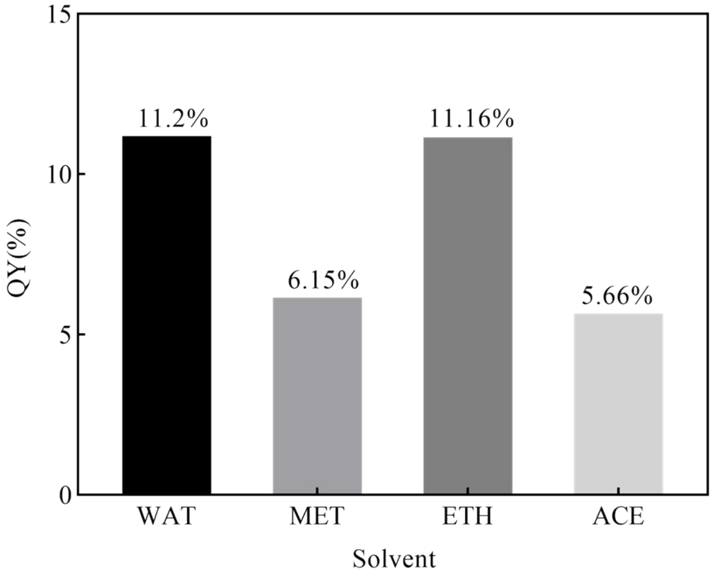

Supplemental Fig. 3 Fluorescence quenching rate (QE) at different B-CQDs concentrations (A), different pH (B), and different detection times (C).

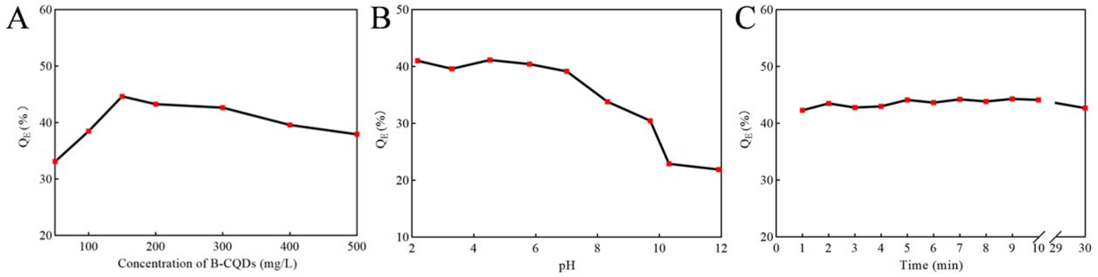

Supplemental Fig. 4 Photographs of B-CQDs solutions under UV light (365 nm) irradiation with different interfering substances and TC. From left to right in the first row: (1) TC, (2) kanamycin sulfate (KAN), (3) streptomycin sulfate (STR), (4) spectinomycin (SPE), (5) gentamycin sulfate (GEN), (6) Zn^{2+}, (7) Ni^{2+}, (8) Na^{+}, (9) Mg^{2+}, (10) K^{+}, (11) Fe^{2+}, (12) Fe^{3+}, (13) Cu^{2+}, (14) Co^{2+}, (15) Ca^{2+}, (16) PO_4^{3-}, (17) NO_3^{-}, (18) Cl^{-}, (19) SO_4^{2-}, (20) CO_3^{2-}.

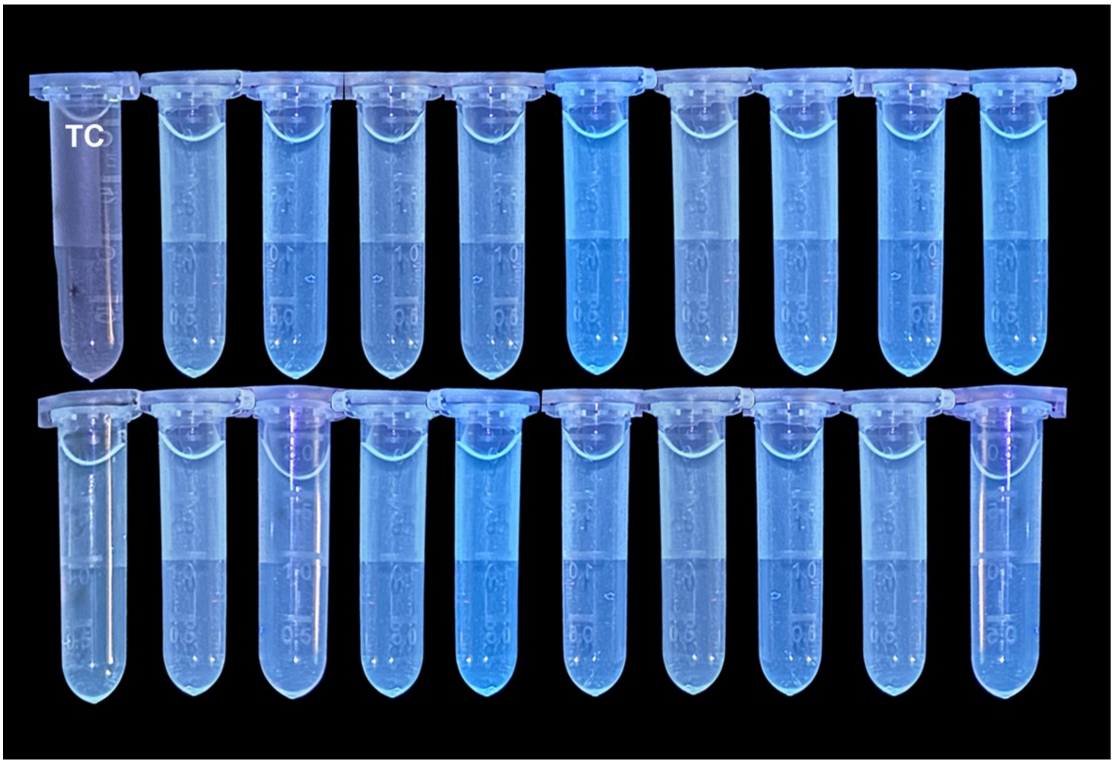

Supplement: RA-016-D5RA07629C-s001 [file RA-016-D5RA07629C-s001.pdf]
